# Liquid Biopsy in Endometrial Cancer: New Opportunities for Personalized Oncology

**DOI:** 10.3390/ijms19082311

**Published:** 2018-08-07

**Authors:** Laura Muinelo-Romay, Carlos Casas-Arozamena, Miguel Abal

**Affiliations:** 1 Liquid Biopsy Analysis Unit, Translational Medical Oncology Group (Oncomet), CIBERONC, Health Research Institute of Santiago de Compostela (IDIS), University Hospital of Santiago de Compostela (SERGAS), Trav. Choupana s/n, 15706 Santiago de Compostela, Spain; laura.muinelo.romay@sergas.es; 2 Translational Medical Oncology Group (Oncomet), CIBERONC, Health Research Institute of Santiago (IDIS), University Hospital of Santiago de Compostela (SERGAS), Trav. Choupana s/n, 15706 Santiago de Compostela, Spain; carlos.casas_95@hotmail.es

**Keywords:** liquid biopsy, uterine aspirate, circulating tumour cells (CTCs), circulating tumour DNA (ctDNA), exosomes

## Abstract

The identification of new molecular targets and biomarkers associated with high risk of recurrence and response to therapy represents one of the main clinical challenges in the management of advanced disease in endometrial cancer. In this sense, the field of liquid biopsy has emerged as a great revolution in oncology and is considered “the way” to reach personalised medicine. In this review, we discuss the promising but already relatively limited advances of liquid biopsy in endometrial cancer compared to other types of tumours like breast, colorectal or prostate cancer. We present recent data analysing circulating tumour material in minimally-invasive blood samples, but also in alternative forms of liquid biopsy like uterine aspirates. Proteomic and genomic studies focused on liquid-based uterine samples are resulting not only in optimal diagnostic tools but also in reliable approaches to address tumour heterogeneity. Likewise, circulating tumour cells (CTCs) and circulating tumour DNA (ctDNA) represent an opportunity for the correct stratification of patients, for the assessment of early recurrent disease or for the real-time monitoring of therapy responses. Appropriately designed studies and implementation in clinical trials will determine the value of liquid biopsy for precision oncology in endometrial cancer.

## 1. Challenges in Endometrial Cancer

Endometrial cancer (EC) is the fourth leading cancer in women from developed countries. In Europe, the number of new cases was about 100,000 in 2012, with an incidence of 13.6 per 100,000 women [1]. This tumour originates in the inner layer of the uterus when epithelial cells lining the myometrium start to proliferate abnormally. Although most ECs are diagnosed early, mainly due to symptomatic postmenopausal metrorrhagia, up to 20% of the lesions progress to a high-stage carcinoma. Unfortunately, the five-year survival in this group of women drops to 15%, compared to 90% in women diagnosed with confined disease. Myometrial infiltration and the appearance of disseminated aggressive tumour cells are crucial events for prognosis and death in EC [2]. Surgery represents the primary treatment. In addition, patients with high risk of recurrence also receive adjuvant radiotherapy, together with chemotherapy that is restricted to metastatic/recurrent disease and high-grade ECs. However, traditional chemotherapy regimens are less effective in comparison with other cancers. This overview situates the clinical challenge on the identification of new molecular targets and biomarkers associated with a high risk of recurrence and/or with response to therapy as valuable tools to improve our management of advanced disease in endometrial cancer.

EC is classified into two distinct groups, type I and type II, which differ in molecular, clinical and histopathological characteristics. Type I tumours are low-grade and estrogen-related endometrioid carcinomas (EEC), while type II are non-endometrioid (NEEC), mainly serous and clear cell carcinomas. To date, this classification has been demonstrated to be an important predictor of survival, but also a determinant for the extent of the initial surgical procedure and subsequent use of adjuvant therapy. However, the molecular heterogeneity associated with the histological diversity of this type of cancer makes the current treatment options insufficiently personalised. To this regard, the integrated genomic, transcriptomic and proteomic characterisation of EC performed by The Cancer Genome Atlas Research Network (TCGA) revealed four groups of tumours [3]. The first group (EEC1) includes EEC with somatic inactivating mutations in POLE exonuclease and very high mutation rates (hypermutated) (7%); it is associated with a good prognosis. The second group (EEC2) includes EEC with microsatellite instability, frequently with MLH-1 promoter hypermethylation and high mutation rates (28%). The third group (EEC3) is composed of EEC with low copy number alterations (39%). Importantly, both the second and third groups show similar progression-free survival rates. Finally, the fourth group (serous-like or copy-number high) (26%) shows low mutation rate but frequent *TP53* mutations. This group has worse prognosis, being predominantly composed of serous carcinomas with some sporadic cases of ECC (mainly EEC3 and some EEC1–2). The incorporation of TCGA surrogate classification into clinical practice should carry important advantages in the management of EC patients [4]. The prognostic value of TCGA in EC has been corroborated in large cohorts included in studies developed by the Vancouver and PORTEC (Post Operative Radiation Therapy in Endometrial Carcinoma) groups [5,6]. This can be especially relevant in adjuvant treatment choices for high to intermediate-risk EC patients that are likely to be impacted by the integrated molecular classification [7], while recurrent disease may continue to represent an additional challenge. Despite these stratification conditionings, and as demonstrated in the majority of solid tumours, EC shows intratumour heterogeneity with different neoplastic cell components within the same tumour. These cells have different morphologic and molecular features that may present a relevant clinical impact, especially for the assessment of prognosis and clinical management of EC patients [8]. In this sense, the use of liquid biopsies to diagnose and characterise EC can facilitate the integration of tumour heterogeneity into the therapy selection and monitoring.

## 2. Liquid Biopsy

Nowadays, research efforts are focused on the discovery of new non-invasive methods for the diagnosis and comprehension of the tumour molecular architecture in real time. In comparison with traditional biopsies, the study of the tumour material present in bodily fluids can provide valuable information for the diagnosis of tumours with low accessibility, or for a more complete overview of tumours in advanced stages where there are different tumour locations to be interrogated. Liquid biopsies also offer advantages to monitor the tumour evolution and the response to therapy with more accuracy than current clinical imaging techniques. In this sense, the field of liquid biopsy has emerged as a great revolution in oncology and is considered “the way” to reach precision medicine. In addition to blood, several other bodily fluids such as saliva, urine, cerebrospinal fluid (CSF), uterine aspirates, pleural effusions or even stool have been shown high interest as a non-invasive source of tumour-derived material [9]. This tumour circulating material is mainly composed of circulating tumour cells (CTCs), circulating tumour DNA (ctDNA), circulating tumour miRNA, proteins and exosomes [10].

The analysis of these different types of liquid biopsy has been successfully applied in oncology research during the last two decades, closely linked to the development of ultrasensitive methods for their detection. In fact, the main limitation to working with liquid biopsy is the low quantity of tumour material present in circulation. For example, in metastatic patients, the mean CTC level is 1 CTC/10^6–8^ mononuclear cells, while ctDNA is normally less than 0.01%. Fortunately, nowadays we have highly sensitive techniques to tackle liquid biopsy analyses with enough guarantees [9].

CTC research is considered the start-point of the liquid biopsy field. Early in the formation and growth of a primary tumour, cells are released into the bloodstream. Several groups are studying the clinical benefit of CTC monitoring. CTCs have been validated as a prognostic marker in metastatic breast cancer and other solid tumours such as prostate, colorectal, and lung cancer, showing even more accuracy than conventional imaging methods for response evaluation. However, there are still technological challenges to use CTC monitoring to detect minimal residual disease in patients at early stages. On the other hand, the molecular characterisation of CTCs is of great value to guide the selection of targeted therapies since it allows clinicians to have a dynamic view of different molecular targets such as ERBB2, EGFR, AR or PD-L1, among others [11,12].

Despite all the studies demonstrating the relevance of CTCs for cancer management, the results from clinical trials have failed to demonstrate a clear clinical benefit. In comparison, the younger brother of CTCs, the ctDNA, has already been implemented in routine clinical practice after EMA (European Medicines Agency) approval of the EGFR mutation test (Therascreen EGFR Plasma, Qiagen) in plasma of patients with non-small cell lung cancer (NSCLC) [13]. Highly sensitive and specific methods have been developed to detect ctDNA, including beads, emulsion, amplification and magnetics based digital PCR (BEAMing) [14], safe sequencing (Safe-Seq) [15], tagged amplicon deep sequencing (TAm-Seq) [16], and digital PCR [17] to detect point mutations or whole-genome sequencing [18]. Using these technologies, several studies have demonstrated that ctDNA may be a useful tool for drug development and for the study of intratumour heterogeneity and clonal evolution in tumours such as breast, colon, melanoma and NSCLC [13]. In gynaecological tumours, and especially for ovarian cancer, TAm-Seq has demonstrated high sensitivity to detect point mutations [16].

On the other hand, the interest in characterising circulating exosomes and miRNAs is continuously increasing. These tumour entities contribute to cancer development and metastasis, and their detection in a variety of biological fluids represents a promising strategy to identify specific biomarkers with diagnostic and prognostic relevance. An additional advantage of circulating exosomes versus CTCs or ctDNA is that these extracellular vesicles can provide higher amounts of tumour material for genetic analyses. Many kits have been commercialised for improved and simplified isolation, such as ExoQuick (System Bioscience). Furthermore, tumour exosome biomarkers such as HSP60 and GPC1 have been described as valuable candidates for colorectal, pancreatic and breast cancer detection. However, these studies on exosomes and miRNA in blood are still quite exploratory and further validation in clinical studies with standardised protocols is mandatory before the routine use of these biomarkers in the clinic [19,20].

## 3. Liquid Biopsy in Endometrial Cancer

**Figure 1:**
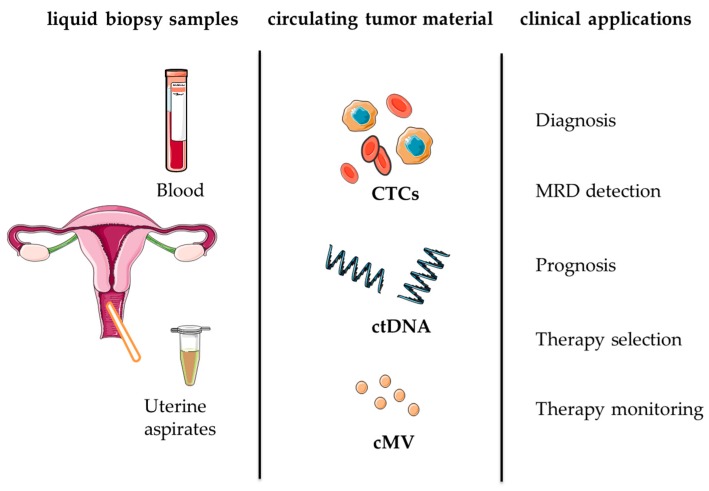
Liquid biopsy for personalised medicine in endometrial cancer. CTCs (circulating tumor cells), ctDNA (circulating tumor DNA), cMV (circulating microvesicles), MRD (minimal residual disease).

Liquid biopsies will play a key role in managing EC patients in the coming years, in addition to multiple other tumour types. This includes different clinical scenarios such as early diagnosis, tumour phenotyping, therapy selection and disease monitoring in real time. Below, we provide an overview of the different forms of liquid biopsy that can be exploited in patients with endometrial cancer towards personalised medicine (Table 1, Figure 1).

### 3.1. Uterine Aspirates

In addition to peripheral blood as the prototypical form of liquid biopsy and main source of tumour material with clinical utility, the uterine aspirate represents an alternative form of liquid biopsy with high relevance in gynaecological malignancies. This is of special importance in EC diagnosis to address an increasing incidence in developed countries [1]. Risk factors for EC include age ≥40 years, obesity, diabetes, hypertension, estrogen using, tamoxifen treatment, and family history of malignant tumours. Many of these factors are tightly linked to current lifestyles in developed countries. An effective screening strategy for women with high-risk factors may contribute to the early detection and management of EC, and a screening policy using liquid-based cytology could be considered in selected high-risk groups of patients in developed countries (Table 1). The diagnostic procedure consists of pelvic examination and transvaginal ultrasonography, followed by the histopathologic observation of an endometrial biopsy, which is preferably obtained by a minimally invasive aspiration from the uterine cavity using a Cornier pipelle (i.e., uterine aspirate or pipelle biopsy). Diagnosis is achieved by the observation of abnormal cells in the uterine aspirate, which presents high sensitivity to detect EC [21]. However, high failure rates with an average of 22% of histologically inadequate specimens have been reported, and a more invasive test such as dilatation and curettage (D&C) or hysteroscopy must be performed, with the added risks of anesthesia, infection and perforation, and higher health care costs. There is a high concordance in molecular subtype assignment between hysterectomy specimens and diagnostic endometrial specimens as obtained by office biopsy (e.g., pipelle) or dilatation and curettage [22]. This makes liquid-based endometrial cytology, which prepares samples for cytology examination by depositing the collected sample into a preservative liquid, a useful method for detecting endometrial pathologies as a first-line approach compared to the more invasive, painful and expensive endometrial biopsy, D&C and hysteroscopy. Cytology sampling performed by brushing the uterus cavity followed by a liquid-based smear has demonstrated an optimal diagnostic accuracy [23].

The endometrial fluid is a non-invasive sample which contains numerous secreted proteins representative of endometrial function and reflects the state of the endometrium. This type of liquid biopsy can result in a comprehensive catalogue of proteins of the endometrial fluid during the secretory phase of the menstrual cycle [24], but also as a promising biological fluid in which to identify potential endometrial cancer biomarkers for its early diagnosis, such as costars family protein ABRACL and phosphoglycerate mutase 2 [25]. The fluid fraction of uterine aspirates are minimally invasive samples with an important value for the screening of EC protein biomarkers, leading to uterine aspirate-based signatures to diagnose EC and classify tumours in the most prevalent histologic subtypes [26]. This will improve diagnosis and assist in the prediction of the optimal surgical treatment. In addition to proteomics in uterine aspirates as an alternative form of liquid biopsy, the potential of targeted genetic sequencing of uterine aspirates has been assessed as a pre-operative tool to obtain reliable information regarding the mutational profile of a given tumour, even in samples that are not histologically classifiable [8]. Notably, the genetic analysis of uterine aspirates captures the high intratumour genetic heterogeneity associated with endometrial cancer, solving the potential problem of incomplete genetic characterisation when a single tumour biopsy is analysed. PapSEEK, a recently developed test that interrogates for mutations in 18 genes as well as for aneuploidy after Tao and Pap brush, showed 81% (95% CI, 76–84%) of EC patients with detectable mutations, including 78% of patients with early-stage disease and 89% of the patients with late-stage disease. In addition, comparing intrauterine sampling with a Tao brush and endocervical sampling with a Pap brush, the former showed an improved detection rate of 93% of 123 (95% CI, 87–97%) patients with endometrial cancer. The most commonly mutated genes in both Tao and Pap brush samples were: *PTEN* (63%), *TP53* (42%), *PIK3CA* (36%), *PIK3R1* (20%), *KRAS* (17%), *CTNNB1* (15%), *FGFR2* (15%), *RNF43* (11%), *PPP2R1A* (7%), *POLE* (7%), and *FBXW7* (6%), clearly representing the endometrial cancer mutational landscape [27].

### 3.2. Circulating Tumour Cells (CTCs)

The presence of circulating tumour cells (CTCs) has been evaluated with the FDA approved technology CellSearch. The findings consistently point to a small number of high-risk EC patients presenting with EpCAM positive CTCs in circulation at the time of diagnosis, although limited cohort studies have been conducted: a study with 7% (*n* = 28) CTC-positive grade 3 EC patients with an association between positive CTCs and both deep myometrial infiltration and positive lymph nodes has been described [28]. Similarly, 15% (*n* = 40) CTC-positive high-risk EC patients have been observed, associated in this case with cervical involvement [29]; in addition, no significant correlation was found between CTCs and serum CA125/HE4 and no CTCs were detected after the first cycle of standard chemotherapy. The ENITEC (European Network for Individualized Treatment in EC) Consortium described a study with 22% (*n* = 32) CTC-positive high-risk EC patients [30]. Finally, another study described 60% (*n* = 30) CTC-positive advanced EC patients with detectable circulating tumour cells, generally associated with non-endometrioid versus endometrioid histology, tumour size ≥5 versus <5 cm, higher-stage disease and worse survival [31]. Although the detection of CTCs in the blood might be of help to determine the potential risk of recurrence in EC patients and to assess the prognosis and possibly guide postoperative treatment, no conclusive information is available, thus limiting their utility in the clinical setting (Table 1).

The combination of isolated CTCs and RTqPCR of a panel of genes has also been explored in EC. Obermayr et al., performed a multimarker analysis using a panel of six genes (*CCNE2*, *DKFZp762E1312*, *EMP2*, *MAL2*, *PPIC*, and *SLC6A8*) for the detection of CTCs which positively identified 64% of a cohort of 25 EC patients [32]. Importantly, gene-expression profiling characterised a strong CTC-plasticity phenotype with stemness and epithelial-to-mesenchymal transition (EMT) features that may provide an advantage in the promotion of metastasis for CTC dissemination and homing. The in vitro recapitulation of this phenotype indicated an improved metastasis efficiency. Moreover, the CTC expression of *CTNNB1*, *STS*, *GDF15*, *RELA*, *RUNX1*, *BRAF* and *PIK3CA* suggested potential therapeutic targets [30]. Regarding biomarkers associated with circulating tumour cells (CTCs), the expression of thyroid transcription factor-1 (TTF-1) correlated with TNM staging, vascular infiltration, and lymphatic metastasis. Progression-free survival (PFS) and the median survival time decreased in the TTF-1-positive group compared with the TTF-1-negative group. Additionally, the recurrence rate increased in the TTF-1-positive group [33].

Finally, using size-based enrichment (MetaCell system), Kolostova et al., demonstrated the feasibility of isolating CTCs from ovarian, endometrial and cervical cancers, and culturing them in vitro for a short time [34]. More recently, and using the same enrichment strategy, peripheral blood samples from 92 patients who underwent a surgical procedure were evaluated for the presence of CTCs, showing an improved detection rate compared to previous studies. In addition, the authors claimed that endometrial CTCs were successfully cultured for further downstream functional and molecular characterisation [42].

In addition to CTCs, other circulating cells have been described as potential EC biomarkers (Table 1). Besides their role in cardiovascular diseases, circulating endothelial cells (CEC) are considered a biomarker for different neoplasms as they play a relevant role in tumour angiogenesis, which is essential for invasive tumour growth and metastasis [43]. Endothelial progenitor cell numbers (CD34, VEGFR2/KDR) in the peripheral blood of women with early endometrial carcinoma were significantly augmented compared with those of healthy control women, while circulating endothelial cell numbers (CD31, CD45) were similar in both groups [35]. By contrast, no prognostic significance or association with clinicopathological features have been demonstrated for the presence of disseminated tumour cells (DTC) in the bone marrow of endometrial carcinoma patients [44,45].

### 3.3. Cell free DNA (cfDNA)

Changes in circulating cell-free DNA (cfDNA) levels have been associated with cancer development and progression. However, very few studies have been developed for evaluating the cfDNA content in EC patients (Table 1). Dobrzycka et al., demonstrated the feasibility of cfDNA detection using the PCR-RFLP and enriched by the PCR-RFPL method in a cohort of 109 patients with EC (87 patients with type I and 22 patients with type II) [36]. In this cohort, *TP53* mutations were also identified in plasma, frequently in early serous carcinomas, and a high frequency of KRAS mutations in grade 2 endometrioid tumours. This was one of the first studies focused on EC that suggested the value of cfDNA monitoring as a marker for predicting the prognosis and selecting individualised treatment regimens [36]. Tanaka et al. also evaluated the cfDNA in 15 healthy individuals, nine with benign gynaecologic diseases, and 53 with EC. They analysed Alu sequences in free DNA fragments by RTqPCR as surrogate markers and found that cfDNA levels in EC tended to be higher than in healthy and benign conditions, although there was no significant difference in cfDNA among stage or histological grade of EC, and no significant changes before and after surgery [37]. A recent report detected augmented levels of total cfDNA and mitochondrial cell-free DNA (cfmtDNA) in serum of patients with EC compared to benign lesions using a SYBR Gold assay and qPCR, respectively. Importantly, they observed that this increase was significantly larger in high grade EC [46]. The same group also explored the cfDNA integrity index as a rapid and noninvasive biomarker that might provide complementary information for diagnosis, prognosis, and treatment stratification in cancer patients [38].

Recently, Zou et al., developed an algorithm called eTumorType to identify different cancer types, including 149 cervical squamous cell carcinoma and endocervical adenocarcinoma and 401 uterine corpus endometrial carcinoma [39]. This test is based on copy number variations (CNVs) of the cancer founding clone, modelling cancer hallmark-associated genes, and integrates cancer hallmark concepts and a few computational techniques. Relatively high accuracies from 0.63 to 0.92 were obtained for these gynaecologic tumours using eTumorType, indicating its value in non-invasive diagnosis [39]. More interestingly, the use of personalised ctDNA biomarkers in gynaecologic cancers including EC could demonstrate the presence of residual tumour; in addition, ctDNA predicted response to treatment in a more dynamic manner relative to currently used serum and imaging studies [40]. Patient/tumour-specific mutations were identified using whole-exome and targeted gene sequencing, and ctDNA levels were quantified using droplet digital PCR. Of particular interest, ctDNA was an independent predictor of survival. Early detection of disease persistence and/or recurrence and the ability to stratify patients in better and worse outcome groups by ctDNA surveillance may improve survival and quality of life in patients with endometrial cancer [40].

Epigenetic markers have also demonstrated great potential for the identification of different tumours. In fact, the methylation status of a number of genes has been described as an accurate tool for cancer detection. For example, SEPT9 showed value for colorectal cancer identification, while MGMT methylation detected brain tumours [47,48]. Margolin et al., described a specific hypermethylation at the ZNF154 CpG island in EC tissues compared to normal controls. These results in tissue were also validated in silico for blood testing [49]. Although these are promising results and methylation markers present advantages in comparison with point mutations, there is still a need for a methodological standardisation to implement these markers in clinical routine.

### 3.4. Circulating Exosomes/miRNAs

Extracellular vesicles containing proteins, lipids, and DNA/RNAs involved in intercellular communication are also considered as alternative forms of liquid biopsy [50]. Among them, exosomes (approximately 100 nm nanovesicles) released from endometrial epithelial cells are an important component of these interactions, as not only are they restricted to tumour cells, but endometrial cancer cells can transmit small regulatory RNAs to endometrial fibroblasts via exosomes [51]. Isolated exosome-like vesicles could become an attractive source of biomarkers, including RNA, by the analysis of the specific inner cargo by RTqPCR from uterine aspirates [52].

For diagnostic purposes, circulating miRNAs (particularly in plasma/serum) have appeared as an important source of clinical material [53]. Torres et al., studied microRNA expression in plasma samples of patients with EC for the first time [54]. They found high expression levels of miR-99a, miR-100 and miR-199b in plasma samples from patients in comparison with healthy controls. The combined analysis for plasma miR-99a/miR-199b resulted in 88% sensitivity and 93% specificity discriminating patients vs. controls, indicating a good diagnostic potential. A more recent study analysed 16 miRNAs in plasma of 34 EC patients and 14 controls, finding miR-9/miR-1228 and miR-9/miR-92a signatures as a good diagnostic tool (Area Under Curve, AUC values ~0.9) [55]. After a genome wide serum miRNA expression analysis, Jia et al., identified miR-222, miR-223, miR-186 and miR-204 up-regulation as a powerful signature for EC detection (AUC of 0.927) [56]. More recently, in a meta-analysis including EC patients, Gao et al., also demonstrated that serum miR-21 could be serve as a novel biomarker for EC. They found higher serum miR-21 levels in patients with benign lesions (*p* = 0.003) and EC (*p* < 0.001) than healthy controls, showing that EC patients also have higher expression levels (*p* < 0.001) than benign lesions [41]. Interestingly, in addition to serum and plasma samples, urinary miRNAs were also explored in patients with EC, finding a specific down-regulation of miR-106b in comparison with healthy donors [57]. All these results evidence a great potential of miRNA signatures in liquid biopsies as valuable information in EC, although until now there is no consistent and clinically validated signature of miRNAs for a reliable clinical management of EC patients.

## 4. Conclusions

Although liquid biopsy can be considered a reality in the clinical setting of some types of cancers, such as breast, colorectal or prostate, it remains as a promising field in gynaecological oncology. The value of liquid biopsy-related technologies in endometrial cancer, such as diagnostic/screening tools based on tumour material in uterine aspirates and as prognostic/monitoring tools based on tumour material in circulation like CTCs or ctDNA (Table 1), needs to be analytically and clinically validated in large clinical trials. Moreover, the potential combination of different and complementary types of liquid biopsy for surgery stratification and during follow-up in intermediate/high-risk and advanced EC patients might result in a comprehensive strategy targeting the identification of mutations for the evaluation of residual disease, the early detection of recurrence, the selection of personalised therapies and disease relapse associated with therapy resistance (Figure 1). These type of strategies appropriately designed and validated in clinical trials, including accurate technical and cost-effective evaluations, represent an opportunity for precision medicine in gynaecological oncology.

## Figures and Tables

**Table 1:** Clinical research approaches to liquid biopsy in endometrial cancer.

Liquid Biopsy	Technology	Clinical Setting	Biomarkers	References
Uterine Aspirate	Targeted Proteomics	Diagnosis	ABRACL and PGAM2; KPYM, MMP9, to identify the disease; CTNB1, XPO2, and CAPG to discriminate between endometrioid endometrial carcinomas (EEC) and serous endometrial carcinoma (SEC)	[25,26]
	Targeted Sequencing	Diagnosis	*PTEN*, *PIK3CA*, *CTNNB1*, *TP53*, *FGFR2*, *KRAS*, *CDKN2A* (most common mutated genes in endometrial cancer (EC))	[8,27]
Circulating Tumour Cells (CTC)	EpCAM-Based Immunoisolation (CellSearch^®^) and IF	Prognosis	CK-8, CK-18, CK-19, *ETV5*, *NOTCH1*, *SNAI1*, *TGFB1*, *ZEB1* and *ZEB2*	[28,29,30,31]
	Density-based Enrichment (Oncoquick) and RTqPCR	Prognosis	*CCNE2*, *DKFZp762E1312*, *EMP2*, *MAL2*, *PPIC*, and *SLC6A8*	[32]
	RTqPCR and flow cytometry	Prognosis	TTF-1 and the mRNA expression of: survivin, β-catenin, miR-15a, and *PTEN*	[33]
	Size-Based Enrichment (Metacell^®^) and Immunodetection	Prognosis	CTCs were defined based on: (i) cell size ≥ 15 μm; (ii) nuclear size ≥ 10 μm); (iii) irregularity of the nuclear contour; (iv) visible cytoplasm; (v) prominent nucleoli; (vi) high nuclear-cytoplasmic ratio; (vii) cluster presence; (viii) mitosis presence.	[34]
Endothelial Progenitor Cells (EPC)	Flow Cytometry	Diagnosis	VEGFR2/KDR and CD34	[35]
Cell Free DNA (cfDNA)	PCR-RFLP	Prognosis	*KRAS*	[36]
	RTqPCR	Prognosis	Alu sequences	[37]
	Alu-RTqPCR	Prognosis	cfDNA content and integrity index	[38]
	NGS	Diagnosis	Copy number variations (CNVs)	[39]
Circulating Tumour DNA (ctDNA)	Droplet Digital PCR	Response to Treatment	Various tumour-specific fusions and mutations in ctDNA	[40]
Circulating miRNA	RTqPCR	Diagnosis/Prognosis	miR-99a/miR-199b, miR-9/miR-1228 and miR-9/miR-92a, miR-222, miR-223, miR-186, miR-204 and miR-21	[38,39,40,41]

## Abbreviations

BEAMing	Beads, Emulsion, Amplification and Magnetics based digital PCR
CEC	Circulating Endothelial Cells
cMV	Circulating Microvesicles
CNVs	Copy Number Variation
CTCs	Circulating Tumour Cells
CK	Cytokeratin
ctDNA	Circulating Tumour DNA
DTC	Disseminated Tumour Cells
D&C	Dilatation and Curettage
EC	Endometrial Carcinoma
EEC	Endometrioid Endometrial Carcinoma
EMA	European Medicines Agency
EMT	Epithelial to Mesenchymal Transition
EPC	Endothelial Progenitor Cells
NEEC	Non-Endometrioid Endometrial Carcinoma
NGS	Next Generation Sequencing
NSCLC	Non-Small Cell Lung Carcinoma
PFS	Progression-free Survival
TCGA	The Cancer Genome Atlas
TAm-Seq	Tagged-amplicon deep sequencing
